# Identification of Novel Loci and Cross-Disorder Pleiotropy Through Multi-Ancestry Genome-Wide Analysis of Alcohol Use Disorder in Over One Million Individuals

**DOI:** 10.21203/rs.3.rs-3755915/v1

**Published:** 2023-12-22

**Authors:** Romain Icick, Alexey Shadrin, Børge Holen, Naz Karadag, Nadine Parker, Kevin O’Connell, Oleksandr Frei, Shahram Bahrami, Margrethe Høegh, Trine Lagerberg, Weiqiu Cheng, Tyler Seibert, Srdjan Djurovic, Anders Dale, Hang Zhou, Howard Edenberg, Joel Gelernter, Olav Smeland, Guy Hindley, Ole Andreassen

**Affiliations:** INSERM; NORMENT, University of Oslo; NORMENT Centre, Institute of Clinical Medicine, University of Oslo and Division of Mental Health and Addiction, Oslo University Hospital, 0407 Oslo; NORMENT Centre, Institute of Clinical Medicine, University of Oslo and Division of Mental Health and Addiction, Oslo University Hospital, 0407 Oslo; NORMENT Centre, Institute of Clinical Medicine, University of Oslo and Division of Mental Health and Addiction, Oslo University Hospital, 0407 Oslo; NORMENT Centre, Institute of Clinical Medicine, University of Oslo and Division of Mental Health and Addiction, Oslo University Hospital, 0407 Oslo; NORMENT, University of Oslo; NORMENT Centre, Institute of Clinical Medicine, University of Oslo and Division of Mental Health and Addiction, Oslo University Hospital, 0407 Oslo; NORMENT Centre, Institute of Clinical Medicine, University of Oslo and Division of Mental Health and Addiction, Oslo University Hospital, 0407 Oslo; NORMENT Centre, Institute of Clinical Medicine, University of Oslo and Division of Mental Health and Addiction, Oslo University Hospital, 0407 Oslo; NORMENT Centre, Institute of Clinical Medicine, University of Oslo and Division of Mental Health and Addiction, Oslo University Hospital, 0407 Oslo; Department of Radiation Medicine and Applied Sciences, Department of Radiology, Department of Bioengineering, University of California, San Diego, La Jolla, CA 92093; Department of Medical Genetics, Oslo University Hospital, Oslo; NORMENT Centre, Department of Clinical Science, University of Bergen, Bergen; Department of Neurosciences, University of California San Diego; Yale School of Medicine; Indiana University School of Medicine; Yale University School of Medicine; NORMENT Centre for Mental Disorders Research, University of Oslo and Oslo University Hospital; NORMENT Centre, Institute of Clinical Medicine, University of Oslo and Division of Mental Health and Addiction, Oslo University Hospital, 0407 Oslo; Oslo University Hospital & Institute of Clinical Medicine, University of Oslo

## Abstract

Alcohol use disorder (AUD) is highly heritable and burdensome worldwide. Genome-wide association studies (GWASs) can provide new evidence regarding the aetiology of AUD. We report a multi-ancestry GWASs across diverse ancestries focusing on a narrow AUD phenotype, using novel statistical tools in a total sample of 1,041,450 individuals [102,079 cases; European, 75,583; African, 20,689 (mostly African-American); Hispanic American, 3,449; East Asian, 2,254; South Asian, 104; descent]. Cross-ancestry functional analyses were performed with European and African samples. Thirty-seven genome-wide significant loci were identified, of which seven were novel for AUD and six for other alcohol phenotypes. Loci were mapped to genes enriched for brain regions relevant for AUD (striatum, hypothalamus, and prefrontal cortex) and potential drug targets (GABAergic, dopaminergic and serotonergic neurons). African-specific analysis yielded a unique pattern of immune-related gene sets. Polygenic overlap and positive genetic correlations showed extensive shared genetic architecture between AUD and both mental and general medical phenotypes, suggesting they are not only complications of alcohol use but also share genetic liability with AUD. Leveraging a cross-ancestry approach allowed identification of novel genetic loci for AUD and underscores the value of multi-ancestry genetic studies. These findings advance our understanding of AUD risk and clinically-relevant comorbidities.

## Introduction

Severe alcohol use disorder (AUD) is a chronic and devastating illness characterized by maladaptive patterns of alcohol use. AUD is common, reaching up to 14% lifetime prevalence in the U.S. (1) and up to 30% in Southern and Eastern African sub-Saharan countries (2). Current treatment options for AUD show modest and variable efficacy (3). Further, AUD leads to several complications, including impaired lipid metabolism, liver and cardiovascular dysfunction, severe psychiatric comorbidity and cognitive impairment, all contributing to the high mortality (4).

AUD is a complex, heritable disorder, with twin heritability estimated at ~ 50% (5). Genome-wide association studies (GWASs) of AUD have begun to elucidate its genetic underpinnings and characterize its polygenic architecture. Most available GWAS findings consistently suggest distinct patterns of genetic correlations for AUD with other mental phenotypes as compared to drinking frequency (6, 7). One of the challenges for genetic studies of alcohol-related traits is genetic heterogeneity (7–9), which makes it critical to map the genetic architecture of clinically-relevant phenotypes with clear definitions. Several previous studies have combined DSM-based AUD case definition with quantitative and/or screening measures from tools such as the AUDIT screening questionnaire. A recent study reported 110 risk loci in a GWAS of problematic alcohol use (PAU) - a relevant proxy for the genetic study of AUD (genetic correlation 85%) (10). These broad definitions have enabled large sample sizes to maximise power for genetic discovery, but capture heterogenous phenotypes. For example, the AUDIT has a two-factor structure - consumption and problems - that is remarkably consistent with its underlying two-factors genetic architecture (11–13). Despite the clear relevance of the problem subscore for genetic studies mixing positively-screened and AUD cases (8, 9), the AUDIT remains a screening tool designed to avoid false-negatives, so that a substantial number of false-positive cases are expected (14). Thus, substantial gaps remain in the knowledge of the the genetic underpinnings of AUD, and how this differs from previously used alcohol-related phenotypes. Yet, AUD represents the most burdensome alcohol-related clinical phenotype for patients and caregivers [AUD is, in part, defined by the distress/burden induced by the disorder (15)].

Multi-ancestry meta-analysis may improve GWAS ability to detect and infer causality and biological relevance of associated genetic variants (16) – including for alcohol-related phenotypes (6–8, 11, 17, 18).

Additionally, cross-disorder post-GWAS analyses have been made standard to explore the shared vs. unique genetic liability to several disorders and traits. This has been particularly relevant for alcohol-related traits, showing how much alcohol (excessive) consumption differs from AUD and PAU in terms of genetic correlation with other mental traits, although they tend to be highly correlated between them. Genetic correlation (r_g_) has been useful in revealing the relationship between AUD and other phenotypes in EUR samples [see, e.g. (6, 11, 13)], with scarce recent findings in AFR samples using recently-developed analytical methods such as POPCORN [see, e.g. (10)]. However, r_g_ remains unable to capture scenarios of a mixture of positive and negative correlations. The MiXeR method is able to characterize overlapping genetic architectures beyond r_g_ in polygenic disorders (19).

The genes mapped to genome-wide significant (GWS) loci in AUD/PAU implicated alcohol metabolism (three alcohol dehydrogenase genes), response to stress (corticotropin releasing hormone and fibroblast growth factor genes), opioid signalling (*OPRM1*) and metal transport (*SLC39A8*). These findings encourage further functional annotations for druggable targets [see, e.g., (20) and (10)] to prioritize subsequent preclinical and clinical research to discover new drugs for AUD. *In silico* functional genomic tools that link loci to genes to expression patterns across specific tissues and cell types can improve the discovery of biological pathways involved in AUD with potential for clinical translation.

We aimed to boost discovery of genetic loci associated with AUD, leveraging novel GWAS data across multiple ancestries and relying on diagnostic criteria for defining AUD cases. We applied novel analytical tools to the multi-ancestry and to ancestry-specific samples to better characterize (i) the genetic architecture of AUD, (ii) its genetic overlap with clinically-relevant mental and general medical traits, disorders and risk factors - to disentangle genetic risk beyond the direct effect of alcohol consumption and (iii) to investigate molecular pathways of AUD including potential druggable targets.

## Methods

### GWAS samples

#### Alcohol Use Disorder.

We extracted summary statistics with p-values and Z-scores from recent GWASs (Table 1) relevant to the AUD phenotype defined according to the DSM-5 or the International Classification of Diseases (ICD) 9/10 (abuse and/or dependence). We included AUD GWASs showing global Linkage Disequilibrium Score Regression (LDSC) genetic correlation (r_g_) > 0.8 with each other (21) (**Supplementary Fig. 1**). Based on these criteria, we selected the following GWAS results for inclusion:

- Million Veteran Program (MVP): ICD 9/10 alcohol abuse/dependence (AUD) - and severe acute intoxication (either one inpatient or two outpatient diagnoses, ICD9 codes 303 to 303.03) (9, 22). All diagnoses were based on validated electronic health records from clinical encounters at settings affiliated with the U.S. Veterans Affairs system. We analyzed MVP AUD data downloaded from the dbGaP website (accession phs001672.v9.p1). Although we did not plan to include alcohol-related traits other than AUD, the summary statistics from the MVP GWAS were not available without intoxication cases, which only represented 0.3% of the sample (N = 226);
- FINNGEN [https://www.finngen.fi/en/access_results, R6 public release (23)]: ICD-9/10 abuse/dependence (AUD) based on validated electronic health records from in- or outpatient care settings in Finland;
- UK Biobank (UKB): ICD 10 abuse/dependence (AUD, see above) based on validated electronic health records from inpatient and primary care settings in United Kingdom (individual level genotypes under accession number 27412);
- Psychiatric Genomics Consortium (PGC) [https://pgc.unc.edu/for-researchers/download-results/ (7)]: DSM-IV alcohol dependence (considered equivalent to severe DSM-5 AUD) diagnosed by trained clinicians’ ratings or semi-structured interviews. These data are not publicly available without the meeta-analysis with the FinnGen sample, and require request to the PGC workgroup.

Combining the samples from all ancestries yielded a total multi-ancestry sample of 1,041,450 individuals, including 102,079 AUD cases (Effective sample size - Neff = 321,343 – see **Supplementary Methods** for calculation). Ancestry-specific sample sizes enabled ancestry-specific analyses for EUR and AFR samples only, as supported by visual examination of the QQ plots (**Supplementary Fig. 2**).

#### GWAS samples for comorbid disorders and traits.

To investigate the genetic architecture of AUD overlapping with mental traits and disorders and with general medical conditions and risk factors which represent frequent comorbidities and complications of alcohol use and AUD (24), we performed cross-disorder analyses using GWAS data downloaded between June 15 and July 1st 2022 (Table 2 & **Supplementary Methods**).

## Statistical analysis

The AUD GWASs included in the meta-analysis were adjusted for sex and the first 6–10 principal components of ancestry. We applied the same procedure to perform our own AUD GWAS in the UK Biobank sample using Regenie (25), which allowed us to keep related individuals for this sample, increasing EUR sample size by 1,408 cases and 70,225 controls (~ 15%). GWAS sample size weighted meta-analysis was performed with METAL (26), with p-values < 5 × 10^−8^ considered genome-wide significant. LD score intercept was calculated using linkage disequilibrium score regression (LDSC) (27). To estimate statistical power and population stratification, QQ plots were produced and genetic inflation factors lambdaGC and lambda1000 were estimated using custom scripts (**Supplementary Fig. 2, Supplementary Table 1**). We also investigated the concordance of findings across ancestries by (i) sign tests assessing the similarity of effect and (ii) testing the Bonferroni-corrected significance of GWS associated loci from the EUR sample in the AFR sample (i.e. (p < 0.05/n EUR significant loci actually found in the AFR sample).

### Definition of genomic loci

Genomic loci were defined using the standard procedure applied in Functional Mapping and Annotation of Genome-Wide Association Studies (FUMA GWAS, https://fuma.ctglab.nl/). Novel loci were identified using a particularly conservative procedure in-house, considering genomic loci +/− 1 kb compared with the GWAScatalog, a curated list of relevant publications, the MRC IEU PheWas tool, and the potential more recent GWASs published since their last updates (**Supplementary Methods**). We report separate novelty assessments for AUD vs. other alcohol-related phenotypes. Of note, for the current study, the novelty checking procedure encompassed GWS loci from Zhou et al.’s PAU (10), and Saunders et al.’s drinking frequency (17).

### Functional annotation

GWS loci were annotated with FUMA (version 1.5.0) by mapping loci to lead SNPs and lead SNPs to credibly mapped genes, defined by at least two converging signals among positional, gene expression and chromatin interaction mapping (FUMA *SNP2GENE*). These genes were linked to cell types using the ad hoc FUMA function (28) within 15 available human tissue types. We included the datasets reporting the largest number of cell types available, refered to as “level 3” in FUMA (217 cell types). We also report the functional impact of candidate SNPs on protein structure, chromatin conformation and tissue-specific gene expression using *ad hoc in silico* databases, as well as the presence of credibly mapped mapped genes as Drugbank hits (FUMA *GENE2FUNCTION*). The full parameters provided to FUMA are available as plain text at the end of the **Supplementary data**, listed as **FUMA parameters 1, 2 and 3 for multi-ancestry, African and European samples,** respectively.

### Quantification of polygenic overlap

We applied univariate MiXeR (29) to estimate SNP-based heritability, discoverability (*i.e*. the average magnitude of additive genetic effects among trait-influencing variants), and polygenicity (*i.e*. the number of trait-influencing variants expected to explain 90% of heritability) of AUD. We used bivariate MiXeR to quantify total polygenic overlap between AUD in EUR and other phenotypes of interest at the genome-wide level (30). To evaluate MiXeR reliability, we reported analyses with Akaike Information Criterion (AIC) differences > 0, as previously reported (19). Due to model fit and use of a European reference genome, MiXeR could only be applied to the EUR sample.

### Cross-disorder genetic correlations across European and African ancestries

Cross-ancestry genetic correlations (r_g_) were estimated between AUD and relevant mental traits and disorders and general medical risk factors and disorders (described above) using *Popcorn* (31).

We applied Bonferonni correction for multiple testing to GWS loci and genetic correlation and False Discovery Rate (FDR, Benjamini-Hochberg) for tissue enrichment. Cell type specificity analyses included both Bonferroni (step one) and FDR (steps two and three).

## Results

### GWAS meta-analysis

The meta-analysis of AUD with the multi-ancestry sample identified 105 genome-wide significant (GWS) risk variants from 37 loci (Fig. 3). We established that seven novel loci for AUD and six for other alcohol-related phenotypes (*e.g*. alcohol consumption, lifetime alcohol use). SNP-based heritability was 0.075 (se = 0.004, p < 1E-17) for the EUR GWAS and 0.053 (se = 0.017, p = 1.4E-3) for the AFR GWAS. Eight loci were unique to the multi-ancestry sample, seven to the EUR sample, which elicited 88 GWS risk variants from 35 loci. In the AFR sample we identified eight GWS risk variants from one locus represented by the lead variant rs1229987, mapped to *RP11-696N14.1*. This variant was in the putative regulatory region of *ADH1B* and in high LD with rs2066702, a functional locus (32) (Fig. 3).

Table 3 shows the location and functional significance of GWS lead variants for AUD for each loci, by ancestry. Credible gene mapping, based on both variant position and *in silico* effect on gene expression or chromatin conformation (see methods section) revealed 55 unique genes mapped to the GWS loci: 41 for multi-ancestry, 40 for EUR and one for AFR. The six loci that were novel for any alcohol-related phenotype, were mapped to *ERI3, BARHL2, SRFBP1* or *LOX, RP11-756H20, CNTLN.* Sixteen locus boundaries were unique to one of the meta-analyses: 9/41 (22%) for the multi-ancestry sample and 7/40 (18%) for EUR sample, leaving 28 locus boundaries that overlapped across multi-ancestry and EUR samples. Nine loci out of the 73 associated with AUD in the three samples had significant heterogeneity in the corresponding meta-analyses (four shared by the multi-ancestry and EUR samples, one in EUR only).

From 27 lead SNPs common to both the EUR and AFR samples, eighteen had a concordant direction of effect in both samples (sign test *p*-value not significant at 0.052, although relatively inconclusive regarding its closeness to reaching significance). Three from the highly significant *ADH1B* locus were replicated in the AFR sample after Bonferroni correction. All these replicated variants had concordant directions of effect compared to the EUR sample. The nine variants with discordant effects in the AFR vs. EUR sample all had *p*-values in the original GWAS > 0.215, making them far from any association with AUD.

### Functional analyses: cells, tissues and gene sets

**Figure 1:**
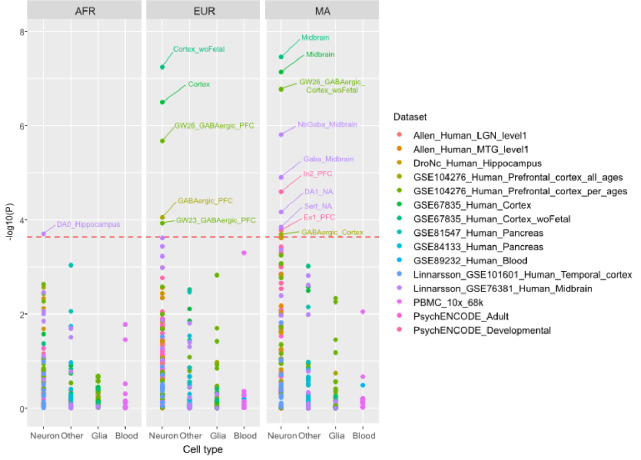
Independent cell types associated with the GWAS meta-analysis results in the African (AFR), European (EUR) and multi-ancestry (MA) samples. Results from FUMA step 3 analysis obtained with 217 Human cell types. woFetal, dataset considered without developing cells; GW, gestation week; PFC, prefrontal cortex; exCA1, hippocampal cornu ammonis excitatory neurons; The complete datasets description is available at https://fuma.ctglab.nl/tutorial#celltype.

**Figure 2:**
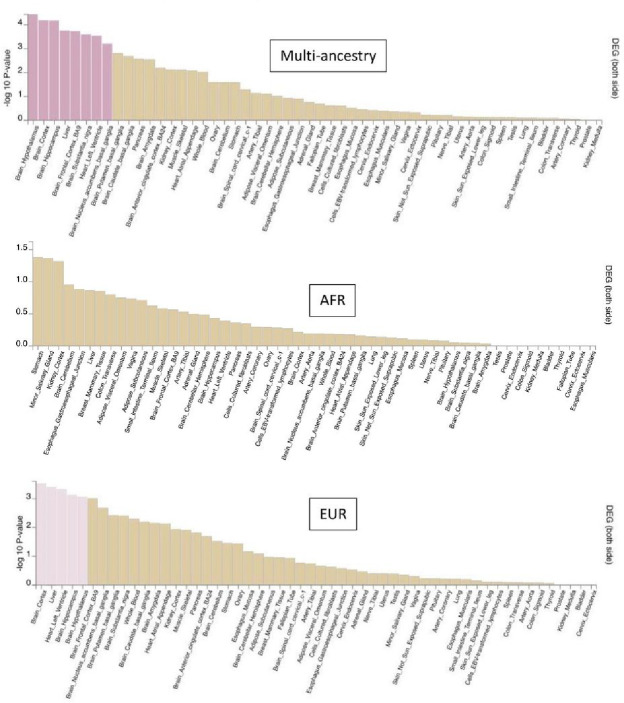
Tissue-specific gene expression enrichment from the multi-ancestry (top panel). African (AFR, middle panel) and European (EUR, bottom panel) analyses. Significant enrichment is represented in pink. (p <0.05 after False discovery rate correction).

**Figure 3:**
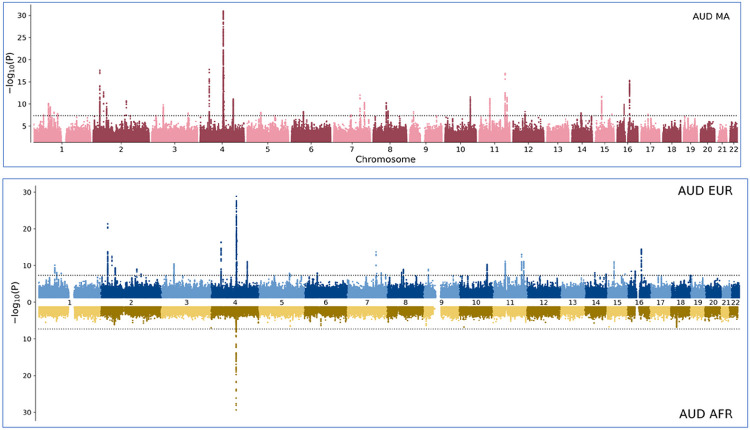
Ancestry Specific Genetic Architecture of AUD in the multi-ancestry analysis (top, red, AUD multi-ancestry) and for the European (top, blue, AUD EUR) and African (bottom, yellow, AUD AFR) samples. −log10 (p-values) obtained by meta-analysis (METAL) are shown on the y-axis while the x-axis represents increasing chromosome numbers from 1 to 22 and positions in K-base pairs. Y-axis is truncated to −log10(P) = 32.

Among 217 cell types tested, ten were signifcantly enriched for genes mapped to lead SNPs in the multi-ancestry sample vs. five in the EUR and one in the AFR sample. (Fig. 1). These cell types mainly included cortical GABAergic neurons, but the multi-ancestry sample also elicited serotonergic and dopaminergic neurons from the adult midbrain and excitatory and inhibitory neurons from the prefrontal cortex. The AFR sample elicited dopaminergic hippocampus neurons. All these cell types remained independently associated with AUD in the multi-ancestry vs. two in EUR (both developmental GABAergic neurons) and none in AFR (see **Supplementary Table 2**). These findings were consistent with tissue enrichment analysis (Fig. 2), where significantly enriched tissues were mostly brain-related (six in the multi-ancestry, three in EUR sample), including the cortex, hippocampus and nucleus accumbens. No tissue was significantly enriched in the AFR sample.

Using MAGMA, there were four significant gene sets in the multi-ancestry analysis, three in EUR, and 14 in AFR (Table 3). All samples were enriched for the alcohol dehydrogenase activity geneset. Compared to EUR, the multi-ancestry sample yielded additional ‘response to alkaloids’ and ‘maintenance_of_presynaptic_active_zone_structure’ gene sets. Interestingly, the AFR analysis elicited nine gene sets related to immunity and inflammation, two to cancer risk, and one to cell aging.

Investigating gene enrichment patterns for Drugbank associations (**Supplementary table 3A-C**) across ancestries, we found 195 unique Drugbank hits in the multi-ancestry analysis, 167 in the EUR analysis and 26 in the AFR analysis. All associations in the AFR and EUR analyses were also found in the multi-ancestry analysis. Twenty-eight findings were unique to the multi-ancestry analysis, including loci related to *CDK5R1* (Cyclin-dependent kinase 5 activator 1 gene), *POR* (cytochrome p450 oxidoreductase) and *DRD2* (Dopamine receptor D2) (**Supplementary Table 3**). Drugbank hits were similar across EUR and AFR ancestries, except for Blood and blood forming organs (Fisher extact test *p* = 0.037) (**Supplementary Table 4**).

### Genetic overlap (MiXeR)

**Figure 4:**
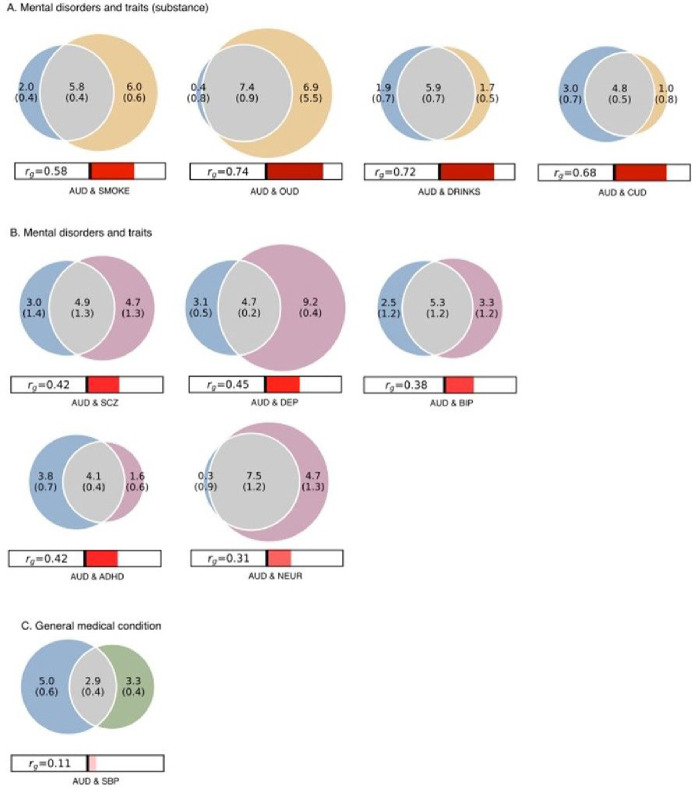
Polygenic overlap between AUD (blue) and clinically relevant phenotypes, after filtering based on estimation of MiXeR stability using the Akaike Informant Criterion. A) CUD, cannabis use disorder; OUD, opioid use disorder; DRINKS, drinks / week; SMOKE, age at smoking initiation (yellow); B) ADHD, attention deficit/hyperactivity disorder; BIP, bipolar disorder; DEP, major depression; SCZ, schizophrenia; NEUR, neuroticism (pink); C) SBP, systolic blood pressure (green).

We obtained reliable MiXeR estimates of polygenicity for age at smoking initiation, drinks / week, neuroticism, CUD, OUD, ADHD, bipolar disorder, major depression, and schizophrenia (*mental traits and disorders*) and systolic blood pressure (*general medical condition*) (Fig. 4). Univariate MiXeR showed that AUD was moderately polygenic (7.8–7.9k ‘causal’ variants), and discoverability was 0.0025 (SD = 0.0002). Bivariate MiXeR showed large overlap between AUD and other substance use phenotypes, sharing 62% of its ‘causal’ variants with CUD, 76% with drinks/week, 95% with OUD and 74% with age at smoking initiation (Fig. 4A). Overlap was substantial, albeit smaller between AUD and non-substance related mental disorders: AUD shared more than half its loci with ADHD (52%), bipolar disorder (53%), major depression (60%) and schizophrenia (62%, Fig. 4B), and mental traits, with a particularly large genetic overlap with neuroticism (96%, Fig. 4C). There was also a large overlap between AUD and systolic blood pressure (37%, Fig. 4C).

### Genetic correlation (r_g_)

**Figure 5:**
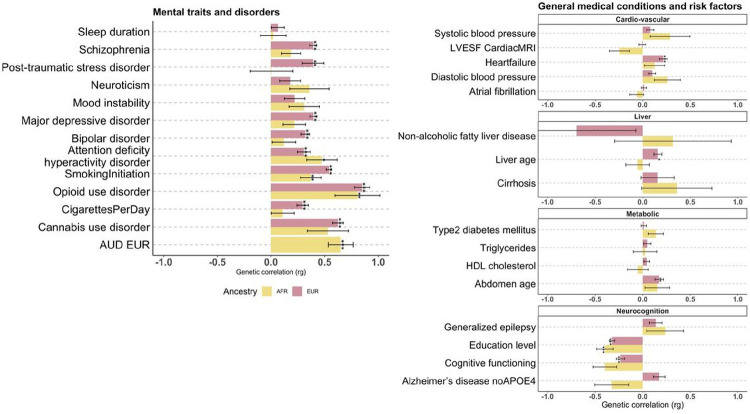
Genetic correlation of AUD with mental traits and disorders (left) and with general medical conditions and risk factors (right), including neuropsychiatric diseases, for the AFR and the EUR samples, separately. The secondary traits are listed. *p <0.05, **p< 0.01, ***p <0.001 (Bonferroni-corrected). LVESF, left ventricular ejection systolic fraction; MRI, magnetic resonance imaging; HDL, hight density lipoproteins; APOE4, Apolipoprotein ε4 locus. AUD EUR, Alcohol Use disorder, European.

The genetic correlation of AUD was moderate between EUR and AFR ancestries (rg=0.65, FDR = 9.3 × 10^−7^). Genetic correlation patterns were overall similar in significance and magnitude across mental traits and disorders (Fig. 5). The strongest correlations were found for ADHD (EUR/AFR r_g_=0.47/0.30), age at smoking initiation (0.54/0.37) and OUD (0.85/0.81); amongst which the highest *p*_corrected_ was 0.047 for ADHD in the AFR sample). The r_g_ between AUD and CUD, major depression, schizophrenia, bipolar disorder and PTSD were only significant in the EUR sample (highest *p*_corrected_ =0.0054 for PTSD). Consistent patterns were also found for cognitive traits (r_g_=−0.23/−0.4) and educational attainment (−0.32/−0.40); highest *p*_corrected_ =3.3 × 10^−4^ for education in the AFR sample. Significant genetic correlation was also observed between AUD and heart failure (r_g_=−0.22, *p*_corrected_=7×10^−6^), liver age (r_g_=0.16, *p*_corrected_=0.011) and abdominal age (r_g_ =−0.17, *p*_corrected_ =5.4×10^−3^) in the EUR samples. The complete r_g_ results are presented in **Supplementary Table 5.**

## Discussion

This multi-ancestry meta-analysis identified 105 genome-wide significant AUD risk variants from 37 independent genomic loci, including seven novel loci for AUD and six novel loci for other alcohol-related traits. Compared to the EUR sample, multi-ancestry meta-analysis resulted in a slight increase in loci discoverability, but a stronger increase in biological diversity, as advocated by, *e.g.*, Saunders et al. (17). AUD loci implicated genes enriched in key brain regions and cells. We confirmed the strong association between *ADH1B* SNPs and AUD in both EUR and AFR samples. We confirmed extensive shared genetic liability between AUD and other substance use phenotypes, mental traits and disorders (especially ADHD and neuroticism) and other medical conditions, consistent with and extending previous work with AUD and PAU phenotypes (6, 7, 9, 13).

To the best of our knowledge, the current study represents the largest GWAS meta-analysis of DSM/ICD-defined AUD, with 1,041,450 participants; identifying ~ 50% more loci than recently published AUD GWASs (6, 9). A multi-ancestry meta-analysis of PAU GWAS data (AUD + AUDIT-problem subscale; N = 1,079,947) identified a total of 110 genetic risk variants across diverse ancestries (10), without providing the specific AUD results. Our study identifies almost as many risk variants, yet focusing on a narrow AUD phenotype. As with the current study, Zhou et al. (10) also provided evidence for the role of the dopamine receptor type 2 gene (*DRD2*), in line with previous studies (6, 33).

Several genes mapped to our novel AUD loci had previously been associated with alcohol-related phenotypes (*e.g.*, drinking quantity or frequency, lifetime alcohol use). This suggests some molecular mechanisms are shared between such alcohol-related phenotypes and AUD. It is noticeable that about half of the AUD loci were related to regulatory regions and not to altered protein structure or function. This supports the role of regulatory mechanisms in the pathophysiology of AUD, as in many other complex phenotypes (34). The single AFR GWS locus was mapped to a long ncRNA, but it likely reflects the strong LD with the functional *ADH1B* variant rs2066702 (0.78–0.98 across 1000 genomes AFR populations).

A key finding in the current study was that ancestral diversity improved the functional mapping, and allowed the discovery of cell types, tissue types and gene sets with potential relevance to the neurobiology of AUD. This was more than expected from the modest increase in the number of GWS loci in the multi-ancestry vs. EUR samples. Since most of the cell types enriched in the multi-ancestry meta-analysis also represent the EUR sample top signal (still not significant), we believe the multi-ancestry meta-analysis shows an actual power increase. First, functional analyses doubled the number and diversity of significantly enriched cell types in multi-ancestry *vs*. EUR samples. These cell types included GABAergic, serotoninergic (*Sert*^+^) and dopaminergic (labelled DA1 in EUR) neurons from the midbrain, dopaminergic neurons (DA0 in AFR) from the hippocampus, and prefrontal neurons. This is consistent with previous evidence supporting the involvement of this brain region and these cell types in AUD, particularly GABAergic neurons in the midbrain in a previous GWAS of maximum alcohol consumption (18) and - more generally - in emotion processing (35) and the action of benzodiazepine medication against alcohol withdrawal syndrome. Secondly, eight tissues were associated with GWS AUD loci in multi-ancestry meta-analysis, 38% more than the EUR sample (N = 5), and mostly included striatal brain regions. Interestingly, the multi-ancestry meta-analysis revealed pathways of relevance to the neuroimaging findings in AUD (36) and to our cell type analysis, including the substantia nigra, frontal cortex and nucleus accumbens. The association with the hypothalamus, a region that regulates liquid intake, could be related to the consumption component of AUD, in line with our previous findings associating hypothalamus with both alcohol consumption and AUD loci (33) and with experimental data regarding altered hypothalamic-pituitary-adrenal axis after chronic alcohol exposure (37). The current findings further illustrate the complementarity of the tissue-level and cell-level bioinformatic approaches to open therapeutic avenues in AUD (38, 39). However, actual experimental biological work is needed to confirm the mechanistic understanding, as in, *e.g*., (40).

The alcohol dehydrogenase genes were enriched in all samples, but the AFR sample showed additional enrichment in several immunity/inflammation-related pathways (14 sets *vs*. four in multi-ancestry and two in EUR), in line with a recent meta-analysis showing associations between cytokine levels and AUD (41). Although such a difference in the number of gene sets in AFR compared to EUR samples may represent a degree of noise in the analysis, MAGMA has shown high detection power with little type I error inflation (42).

The current sample size was large enough to apply MiXeR to a wider range of traits and disorders than in our previous work on AUD (33). We provide novel evidence that substance use phenotypes are highly polygenic, and estimated the polygenicity of AUD to 7.2k – 8.5k causal variants. We recently showed that other mental disorders have similar polygenicities, ranging between 5.6k (ADHD) and 14.5k (major depression) causal variants (19). This high polygenicity could partly explain the high level of comorbidity in AUD given the extensive genetic overlap between AUD and other substance use and mental disorders (notably ADHD, bipolar disorder, major depression and schizophrenia; Fig. 5). The particularly large overlap between AUD and neuroticism (92%) - even in the absence of significant genetic correlation – further supports the hypothesis that the shared genetic component of mental disorders also includes AUD (19) and partly relies on the shared liability to neuroticism. Overlapping variants seem to exert bidirectional effects, both increasing or decreasing the risk across phenotypes. More GWAS data, especially from non-EUR samples, are needed to reliably estimate the overlap between AUD and general medical conditions. Still, we report substantial shared polygenicity between AUD and systolic blood pressure (36%) in the absence of significant genetic correlation.

There were mostly consistent patterns of genetic correlations across ancestries, especially regarding AUD and mental traits and disorders, especially OUD - significantly extending recent work in the AFR population (10). Of particular interest were the correlations between AUD and conditions that are usually attributed to the toxic effects of alcohol (*e.g*., MRI-predicted abdominal age and heart failure). This suggests a more mixed picture of highly probable toxic alcohol effects and shared molecular underpinnings. We plan to compare these correlation patterns by using individual-level genetic and phenotypic data regarding alcohol consumption vs. AUD in the near future.

Our study has limitations. There was insufficient statistical power in several cross-disorder analyses involving AUD, calling for urgent action to gather more genetic data in non-EUR populations. The effect direction across EUR and AFR ancestries was concordant in 2/3 of GWS loci, which may limit the reliability of some aspects of the multi-ancestry meta-analysis. However, this particular analysis had strong statistical power. Some *in silico* data used for downstream analyses are sourced from EUR samples only. Additionally, although the reference SNP and LD maps from the 1000 genomes project are sourced from relatively diverse non-EUR samples, AUD cases of AFR ancestry were recruited in Western countries (US, notably) and no data was available in terms of genetic heterogeneity for these particular samples. Same applies to *FUMA* analyses, for which the detail regarding LD structure is higher for EUR than for other ancestries. Overall, caution is thus advised when interpreting findings for functional or cross-ancestry analyses. Finally, the almost exclusive use of summary statistics, preventing us from performing subgroup analyses that could help to identify clinical subgroups and control for potential mediating factors. However, this remains the only way to leverage very large GWAS samples to date, especially for case-control analyses.

The current study leveraged multi-ancestry samples to discover several novel AUD risk loci and improve the biological diversity of associated molecular pathways, cell types and brain regions implicated in AUD.

## Figures and Tables

**Table 1:** GWAS data, including ancestry breakdown. EUR, European; AFR, African; HA, Hispanic American; EAS, East Asian; SAS, South Asian; AUD, alcohol Use Disorder; ICD, International Classification of Diseases; DSM, Diagnostic and Statistical Manual of Mental Disorders; GWAS, Genome-wide association studies; MVP, Million Veteran Program; PGC, Psychiatric Genomic Consortium; SNP, Single nucleotide polymorphism. Neff, effective sample size

GWAS	Phenotype	Sample size (cases)	Sample size (cases) by ancestry (n)	Age (years), sex (%)	Maximum number of SNPs (n, millions)
*MVP*	AUD (+ severe acute intoxication) (ICD-9/10)	305,511 (68,913)	EUR: 221,137 (45,943) AFR: 56,648 (17,267) HA: 14,175 (3,449) EAS: 13,551 (2,254)	30–75 years 8% women	6.8
*UK Biobank*	Alcohol abuse / dependence (ICD 10)	425,224 (8,201)	EUR: 409,558 (7,910) AFR: 7,045 (87) SAS: 8,621 (104)	30–69 years 56% women	9.9
*FinnGen*	AUD (ICD-9/10)	260,405 (10,688)	EUR: 260,405 (10,688)	Median 63 years 56% women (total sample*)	20.2
*PGC without FinnGen*	Alcohol dependence (DSM-IV)	50,310 (14,377)	EUR: 44,030 (11,042) AFR: 6,280 (3,335)	39% women > 18 years Pooled mean 34.7 from 30% of the sample**	10.9
*Total (multi-ancestry)*	AUD (+ severe acute intoxication)	**1,041,450 (102,179)** *Neff =* 321,343	**• EUR: 935,130 (75,583)** *Total Neff =* 249,626 (Finngen, 40,997; PGC without Finngen, 25,267; MVP 152,333; UKB, 31,029) **• AFR: 70,060 (20,689)** *Total Neff =* 53,350 (MVP 48,015; PGC without Finngen, 4,991; UKB, 343,755) **• HA: 14,175 (3,449)** *Total Neff =*10,439 (MVP) **• EAS + SAS: 21,972 (2,358)** *Neff =* EAS 7,516 (MVP), SAS 411 (UKB)	*Pooled:* Mean age. 51.8 Women. 32%	Multi-ancestry: 24.8 EUR: 19.1 AFR: 9.9

* Individuals suffering from AUD are expected to be more often males; median age is less relevant for the FINNGEN cohort compared to the others since all individuals are followed-up from birth.

** Pooled mean age was obtained using the cohorts mean ages, except for the PGC sample, where it was extrapolated from the mean age of the Pale-Yenn and SAGE cohorts, which represent ~ 30% of the total PGC sample - yielding pooled mean age = 34.7 based on PMID 27028160.

**Table 2:** phenotypes and corresponding reference, of which summary statistics for cross-disorder analyses were acquired.

Phenotypes	Reference

*Mental traits and disorders*	

Mood instability	Ward 2020, PMID: 29187730
Neuroticism	Nagel 2020, PMID: 29942085
Sleep duration	Dashti 2019, PMID: 30846698
Cigarettes per day	Liu 2019, PMID: 30643251
Smoking initiation	Liu 2019, PMID: 30643251
Drinks/week	Liu 2019, PMID: 30643251
Cannabis use disorder (cud)	Johnson 2020, PMID: 33096046
Opioid use disorder (oud)	Kember 2022, PMID: 36171425
Attention deficit/hyperactivity disorder (adhd)	Demontis 2023, PMID: 36702997
Bipolar disorder	Mullins 2021, PMID: 34002096
Major depression	Howard 2021, PMID: 30718901
Post-traumatic stress disorder (ptsd)	Nievergelt 2022, PMID: 31594949
Schizophrenia	Trubetskoy 2022, PMID: 35396580

*Cognitive traits and neurological disorders*	

Cognitive functioning	Savage 2018, PMID: 29942086
Education level	Okbay 2022, PMID: 35361970
Alzheimer’s disease	Wightman 2021, PMID: 34493870
Generalized epilepsy	Abou-Khalil 2018, PMID: 30531953

*General medical diseases and risk factors*	

High density lipoprotein (HDL) cholesterol	Graham 2021, PMID: 37237109
Triglycerides	Graham 2021, PMID: 37237109
Type 2 diabetes mellitus, non-alcoholic fatty liver disease	Mahajan 2022, PMID: 35551307
Liver cirrhosis	Sveinbjornsson 2022, PMID: 36280732
Liver age	Sveinbjornsson 2022, PMID: 36280732
Abdominal age (whole-body MRI)	Sveinbjornsson 2022, PMID: 36280732
Diastolic blood pressure	Le Goallec 2022, PMID: 35418184
Systolic blood pressure	Evangelou 2018, PMID: 30224653
Atrial fibrillation	Nielsen 2018, PMID: 30061737
heart failure	Shah 2020, PMID: 31919418
left ventricule ejection systolic fraction	Pirrucello 2020, PMID: 32382064

**Table 3:** Genome-wide significant loci in the multi-ancestry, European (EUR), and African (AFR) samples (genome build GRCh37.p13). uniqID is chromosome:position (base pairs):alternate allele:reference allele. rsID represents the SNP ID according to the reference database dbSNP. CADD, Combined Annotation Dependent Depletion (non-coding variants; RDB, regulomeDB score (not all variants could be found in this database, as indicated by blank cells). POLYPHEN estimates the tolerability/deleteriousness of exonic variants only. Credibly mapped genes are considered when designated by two out of positional/expression/chromatin interaction mapping analyses.

uniqID	rsID	Chromosome	Position (bp)	GWAS *p*	*Heterogeneity estimates*		Credibly mapped gene	CADD/POLYPHEN	RDB	func
					*HetISq*	*HetPVal*				
*multi-ancestry*										
1:44776240:A:C	rs12026967^b,c^	1	44776240	3.11E-08	13.2	0.3212	*ERI3*	0.762	3a	intronic
1:66441965:C:T	rs61799435	1	66441965	9.07E-11	10.6	0.3448	*PDE4B*	0.063	5	intronic
1:73882478:A:G	rs1475064	1	73882478	5.02E-10	0	0.8706	*RP4–598G3.1*	5.795	7	intergenic
1:91205831:C:T	rs10922911^a,b^	1	91205831	8.77E-09	0	0.8769	*BARHL2*	0.487	7	intergenic
1:106723404:C:T	rs12044479^a,b,c^	1	106723404	1.81E-08	0	0.9506	*RP5–947P14.1*	0.734	5	intergenic
2:27741237:C:T	rs780094	2	27741237	2.52E-18	68.2	0.0008541	*GCKR*	1.852	2c	intronic
2:45141180:C:T	rs494904	2	45141180	2.02E-13	0	0.8534	*RP11–89K21.1*	4.063	7	intergenic
2:57987593:C:T	rs11682175	2	57987593	7.74E-11	0	0.8476	*CTD-2026C7.1*	0.995	3a	ncRNA_intronic
2:138262443:C:T	rs3748877^c^	2	138262443	4.25E-08	0	0.9887	*THSD7B*	5.598	NA	intronic
2:144225215:A:C	rs13024996	2	144225215	2.12E-11	0	0.9167	*ARHGAP15, AC096558.1, RP11–570L15.2*	1.13	7	ncRNA_intronic
2:161866028:A:G	rs57577502^a^	2	161866028	4.78E-08	52.3	0.02644	*AC009313.2*	7.845	7	intergenic
3:16852736:A:G	rs7625233^b,c^	3	16852736	4.56E-08	0	0.5422	*PLCL2*	9.089	5	
3:49357427:A:G	rs11720542^a^	3	49357427	1.75E-10	22.5	0.2361	*USP4*	5.767	7	intronic
3:157902975:C:T	rs2693546^c^	3	157902975	1.3E-08	0	0.5616	*RSRC1*	2.797	NA	intronic
4:39425248:A:G	rs13146907	4	39425248	1.68E-18	0	0.4349	*KLB*	3.426	6	intronic
4:100239319:C:T	rs1229984	4	100239319	7.9E-160	91.6	4.31E-19	*ADH1B*	13.86	NA	Exonic
4:103198082:A:G	rs13135092	4	103198082	4.25E-17	63.2	0.008096	*SLC39A8*	10.31	6	intronic
4:143873294:G:T	rs2874918	4	143873294	7.95E-12	18.2	0.2809	*RP11–284M14.1*	0.497	6	ncRNA_intronic
5:60091556:A:G	rs6894750^c^	5	60091556	9.46E-09	0	0.4406	*ELOVL7*	0.314	7	intronic
6:51452051:A:C	rs1961821	6	51452051	5.68E-09	0	0.9273	*RP3–335N17.2*	2.615	NA	intergenic
7:75615006:C:T	rs1057868^c^	7	75615006	3.42E-08	0	0.5194	*POR*	13.88	4	Exonic
7:114948351:A:G	rs10270358	7	114948351	1.11E-12	41.1	0.08357	*AC068610.5*	4.102	6	intergenic
7:135100476:C:T	rs2551777^a^	7	135100476	5.03E-11	15.6	0.2994	*CNOT4*	1.15	NA	intronic
8:57424303:A:G	rs35500854^a^	8	57424303	6.11E-11	0	0.5382	*RP11–17A4.2, LINC00968*	5.893	5	ncRNA_intronic
8:64949682:C:T	rs1899899	8	64949682	4.02E-09	0	0.6229	*RP11-32K4.1*	1.455	NA	ncRNA_intronic
9:17260185:G:T	rs11543973b	9	17260185	6.73E-09	0	0.6187	*CNTLN*	2.068	6	intronic
10:110497101:C:T	rs7906104	10	110497101	2.89E-12	0	0.9164	*RP11-655H13.2*	1.592	6	ncRNA_intronic
11:47419129:A:G	rs7924485	11	47419129	6.45E-12	0	0.5938	*RP11-750H9.5*	3.529	7	ncRNA_intronic
11:112849405:A:G	rs4430547	11	112849405	2.18E-08	0	0.4614	*NCAM1*	9.64	7	intronic
11:113436072:A:G	rs7125588	11	113436072	1.25E-17	3.7	0.4063	*DRD2*	1.64	7	intergenic
11:121634608:G:T	rs4936651^a^	11	121634608	3.56E-12	0	0.8606	*SORL1*	0.614	4	intergenic
12:51895882:C:T	rs10876188^c^	12	51895882	5.65E-09	8.5	0.3643	*SLC4A8*	0.433	6	intronic
14:58766617:A:G	rs1957038	14	58766617	1.23E-08	0	0.7072	*ARID4A*	9.588	4	intronic
15:47681367:A:G	rs8034190	15	47681367	2.23E-12	0	0.6417	*SEMA6D, CTD-2050N2.1*	2.426	7	ncRNA_intronic
16:30082508:G:T	rs7201518	16	30082508	1.46E-10	11	0.3414	*ALDOA*	0.119	4	downstream
16:53800954:C:T	rs1421085	16	53800954	5.08E-16	33	0.1442	*FTO*	21.4	5	intronic
17:30626242:A:C	rs11653646^c^	17	30626242	2.42E-08	0	0.9948	*RHBDL3*	14.27	4	intronic
			*EUR*							
1:66440096:C:T	rs2310819	1	66440096	8.29E-11	0	0.7927	*PDE4B*	3.026	6	intronic
1:73873424:A:G	rs2340405	1	73873424	7.48E-09	0	0.7348	*RP4–598G3.1*	0.14	6	intergenic
1:91208451:C:T	rs2166171^a,b^	1	91208451	1.42E-08	0	0.6526	*BARHL2*	0.765	6	intergenic
2:27730940:C:T	rs1260326	2	27730940	4.28E-22	78	0.003446	*GCKR*	13.22	5	Exonic
2:45141180:C:T	rs494904	2	45141180	3.34E-13	0	0.491	*RP11-89K21.1*	4.063	7	intergenic
2:58046683:A:G	rs2717054	2	58046683	4.45E-10	48.2	0.1219	*CTD-2026C7.1*	3.04	NA	intergenic
2:144215811:C:T	rs13411140	2	144215811	9.56E-10	0	0.5234	*ARHGAP15, AC096558.1, RP11–570L15.2*	7.405	5	ncRNA_intronic
2:161865998:A:G	rs57761252^a^	2	161865998	2.39E-08	57	0.07282	*AC009313.2*	0.082	7	intergenic
3:49369383:C:T	rs6809204	3	49369383	3.76E-11	56	0.07783	*USP4*	0.554	6	intronic
4:39425248:A:G	rs13146907	4	39425248	4.06E-17	36.4	0.1937	*KLB*	3.426	6	intronic
4:100239319:C:T	rs1229984	4	100239319	1.7E-141	88.6	7.81E-06	*ADH1B*	13.86	NA	Exonic
4:103198082:A:G	rs13135092	4	103198082	1.31E-17	67.9	0.02508	*SLC39A8*	10.31	6	intronic
4:143865906:A:G	rs4690738	4	143865906	8.04E-12	0	0.5332	*RP11-284M14.1*	0.301	4	ncRNA_intronic
5:121402597:C:T	rs10053942^a,b,c^	5	121402597	1.43E-08	0	0.6714	*SRFBP1, LOX*	1.931	7	intronic
5:124850358:A:G	rs331751b^a,b,c^	5	124850358	3.34E-08	45.4	0.1602	*RP11-756H20.1*	1.83	NA	ncRNA_intronic
6:51461219:G:T	rs2784239	6	51461219	1.27E-08	0	0.9576	*RP3–335N17.2*	0.878	7	intergenic
7:114948351:A:G	rs10270358	7	114948351	1.83E-14	0	0.7057	*AC068610.5*	4.102	6	intergenic
7:117588647:A:G	rs13221279^a,c^	7	117588647	1.6E-08	0	0.4745	*AC003084.2*	3.358	5	intergenic
7:135100476:C:T	rs2551777^a^	7	135100476	1.1E-08	64.2	0.03876	*CNOT4*	1.15	NA	intronic
7:153489530:A:G	rs2533199^c^	7	153489530	3.27E-08	10.9	0.3383	*DPP6*	2.815	NA	intergenic
8:57424303:A:G	rs386725692	8	57424303	6.72E-09	0	0.4427	*RP11–17A4.2, LINC00968*	5.893	5	ncRNA_intronic
8:64956228:C:T	rs1822717	8	64956228	1.11E-09	0	0.5455	*RP11-32K4.1*	0.127	5	ncRNA_intronic
9:17260185:G:T	rs386725693^a,b^	9	17260185	1.11E-09	0	0.8199	*CNTLN*	2.068	6	intronic
10:110565868:C:T	rs7073987	10	110565868	5.65E-11	0	0.7607	*RP11-655H13.2*	1.447	6	ncRNA_intronic
11:47406592:C:T	rs11039216	11	47406592	6.78E-12	68.6	0.02281	*RP11-750H9.5*	0.987	6	ncRNA_intronic
11:112878254:A:C	rs7118907	11	112878254	4.29E-08	0	0.7705	*NCAM1*	3.744	7	intronic
11:113443753:C:T	rs6589386	11	113443753	9.72E-14	0	0.6767	*DRD2*	0.99	7	intergenic
11:121634608:G:T	rs4936651	11	121634608	7.48E-12	0	0.8423	*SORL1*	0.614	4	intergenic
14:58766617:A:G	rs1957038	14	58766617	8.7E-09	0	0.9509	*ARID4A*	9.588	4	intronic
14:104264857:A:G	rs7147171^c^	14	104264857	2.58E-08	9.8	0.3441	*PPP1R13B*	5.333	6	intronic
15:47681367:A:G	rs8034190	15	47681367	8.5E-12	16.4	0.3023	*SEMA6D, CTD 2050N2.1*	2.426	7	ncRNA_intronic
16:13580639:A:G	rs11859355^c^	16	13580639	3.71E-09	61.9	0.0725	*U91319.1*	1.49	6	ncRNA_intronic
16:30092048:A:G	rs9932605	16	30092048	3.27E-09	48.8	0.1188	*PPP4C*	0.328	5	intronic
16:53809247:A:G	rs1121980	16	53809247	3.51E-15	44.4	0.145	*FTO*	0.64	4	intronic
16:54629889:C:T	rs1151284^c^	16	54629889	3.25E-08	0	0.8035	*AC079412.1*	5.333	5	intergenic
			*AFR*							
4:100217127:C:T	rs1229987	4	100217127	6.9E-25	54.2	0.1129	*RP11-696N14.1*	2.636	6	ncRNA_intronic

a Novel loci for AUD,

b Novel loci for other alcohol-related phenotypes,

c Locus unique to the sample. Ten genes were identified in multi-ancestry only (RP5-947P14, ERI3, SLC4A8, ALDOA, RHBDL3, THSD7B, RSRC1, PLCL2, ELOVL7, POR), eight in EUR only (PPP1R13B, U91319, PPP4C, AC079412, SRFBP1 or LOX, RP11-756H20, AC003084, DPP6).

**Table 4:** Significant gene sets associated with AUD loci according to MAGMA analysis in the GWASs with multi-ancestry, European (EUR) and African (AFR) samples. FDR, false discovery rate after Bejamini-Hochberg correction.

Gene set name	Number of genes	FDR
*Multi-ancestry*		
GO_mf:go_alcohol_dehydrogenase_activity_zinc_dependent	6	1.07 × 10^−7^
GO_mf:go_alcohol_dehydrogenase_nad_activity	8	5.54 × 10^−4^
GO_bp: go_response_to_alkaloid	106	9.06 × 10^−3^
GO_bp:go_maintenance_of_presynaptic_active_zone_structure	5	2.15 × 10^−2^
*EUR*		
GO_bp:go_oxidative_rna_demethylation	5	8.26 × 10^−3^
GO_mf:go_alcohoLdehydrogenase_activity_zinc_dependent	6	8.52 × 10^−3^
GO_mf:go_oxidative_rna_demethylase_activity	5	8.26 × 10^−3^
*AFR*		
GO_bp:go_positive_regulation_of_activation_of_janus_kinase_activity	7	8.93 × 10^−5^
GO_mf:go_alcohol_dehydrogenase_nad_activity	8	2.22 × 10^−4^
Curated_gene_sets:galindo_immune_response_to_enterotoxin	73	5.51 × 10^−4^
GO_bp:go_regulation_of_activation_of_janus_kinase_activity	9	8.03 × 10^−4^
GO_bp:go_regulation_of_chemokine_mediated_signaling_pathway	8	2.07 × 10^−3^
GO_bp:go_negative_regulation_of_chemokine_mediated_signaling_pathway	5	5.44 × 10^−3^
Curated_gene_sets:hoffman_clock_targets_dn	9	7.69 × 10^−3^
GO_mf:go_alcohol_dehydrogenase_activity_zinc_dependent	6	9.97 × 10^−3^
GO_bp:go_positive_regulation_of_natural_killer_cell_chemotaxis	5	1.04 × 10^−2^
GO_mf:go_ccr1_chemokine_receptor_binding	4	1.20 × 10^−2^
Curated_gene_sets:ju_aging_terc_targets_dn	6	1.50 × 10^−2^
Curated_gene_sets:reactome_cytokine_signaling_in_immune_system	798	2.16 × 10^−2^
Curated_gene_sets:soucek_myc_targets	8	2.41 × 10^−2^
GO_bp:go_regulation_of_chronic_inflammatory_response	8	2.50 × 10^−2^
